# Scalable Identification of Clinically Relevant Chronic Obstructive Pulmonary Disease Documents in Large-Scale Electronic Health Record Datasets With a Lightweight Natural Language Processing Model: Retrospective Cohort Study

**DOI:** 10.2196/84326

**Published:** 2026-05-12

**Authors:** Mohammed Al-Garadi, Sharon E Davis, Michael E Matheny, Dax Westerman, Adrienne K Conger, Bradley W Richmond, Thomas A Lasko, Iben M Ricket, Laura M Paulin, Jeremiah R Brown, Ruth M Reeves

**Affiliations:** 1 Department of Biomedical Informatics, Vanderbilt University Medical Center, 2525 West End Ave, Suite 1475, Nashville, TN, 37203, United States, 1 (615) 936-6867; 2 Department of Medicine, Vanderbilt University Medical Center, Nashville, TN, United States; 3 Department of Biostatistics, Vanderbilt University Medical Center, Nashville, TN, United States; 4 Tennessee Valley Healthcare System, Department of Veterans Affairs, Nashville, TN, United States; 5 Division of Allergy, Pulmonology, and Critical Care Medicine, Vanderbilt University Medical Center, Nashville, TN, United States; 6 Department of Computer Science, Vanderbilt University, Nashville, TN, United States; 7 Department of Epidemiology, Dartmouth Geisel School of Medicine, Lebanon, NH, United States; 8 Department of Pulmonology, Dartmouth Hitchcock Medical Center, Lebanon, NH, United States; 9 Center for Implementation Science, Dartmouth Geisel School of Medicine, Lebanon, NH, United States

**Keywords:** natural language processing, chronic obstructive pulmonary disease, electronic health record, machine learning, data mining, artificial intelligence, AI

## Abstract

**Background:**

The widespread adoption of electronic health records has resulted in the generation of large volumes of clinical notes. Learning algorithms and large language models can be trained on these resources, but they are susceptible to noise—irrelevant or noninformative data. This sensitivity can lead to significant challenges, including performance degradation and the generation of inaccurate predictions or “hallucinations.” This study addresses a critical challenge in clinical informatics: efficiently filtering millions of documents for relevance before advanced language model processing, particularly in resource-constrained environments.

**Objective:**

We present a novel framework for determining document relevance in clinical settings using a chronic obstructive pulmonary disease (COPD) dataset.

**Methods:**

We developed a novel framework using weak supervision and domain-expert heuristics to generate “silver standard” labels for training data and gold standard expert-annotated labels, creating 2 datasets to optimize the model during the development phase and subsequent testing phase. Various text representation techniques (bag of words, term frequency–inverse document frequency, lightweight document embeddings, compression-based features, and Unified Medical Language System concept extraction) were evaluated. These representations were used to train random forest, extreme gradient boosting, and k-nearest neighbor classifiers. Models were optimized on a small expert-annotated dataset and evaluated on a held-out test set.

**Results:**

The combination of lightweight document embedding with a random forest classifier demonstrated the best performance, achieving a precision of 0.73, recall of 0.86, and *F*_1_-score of 0.80 (95% CI 0.76-0.87) for identifying relevant COPD documents. This significantly outperformed baseline heuristics (precision=0.70; recall=0.38; *F*_1_-score=0.50, 95% CI 0.43-0.56) and other tested methods.

**Conclusions:**

Our study presents a novel framework for identifying COPD-relevant clinical documents using lightweight embedding and machine learning. This approach effectively filters pertinent documents, enhancing information retrieval precision. The framework’s scalability and minimal annotation needs make it promising for diverse health care applications, potentially optimizing clinical outcomes through efficient document selection for data-driven decision support systems.

## Introduction

### Background

Chronic obstructive pulmonary disease (COPD) is a leading cause of mortality in the United States, impacting an estimated 24 million people nationwide [1]. COPD exacerbation–related hospitalizations also incur substantial health care costs, ranging from US $7000 to US $39,200 per hospitalization [2]. Overall, COPD-attributable care amounted to US $49 billion in 2020, with hospitalization comprising approximately half of this total cost [3].

Artificial intelligence (AI) is transforming health care by enhancing the analysis, interpretation, and prediction of medical data. This technological advancement supports clinical decision-making and enables more efficient allocation of limited resources for chronic disease management, particularly for high-risk patients [4,5]. While narrative clinical text is information rich, its unstructured nature poses challenges for direct use in AI-driven clinical decision support systems. To address this, information extraction and representation methods have emerged, leveraging large language models (LLMs) and deep learning techniques [6-10]. These approaches aim to transform raw clinical narratives into structured, machine-readable data. However, significant challenges persist when processing vast document collections. Information relevant to a specific task may be dispersed across only a small fraction of the total corpus, making it inefficient to apply computationally intense LLMs to the entire dataset [11]. Sophisticated preprocessing, filtering, and prioritization algorithms can identify and extract relevant documents, reducing the volume of text that needs to be processed at computationally intense stages of the pipeline [12].

### Weak Supervision for Developing Automated Natural Language Processing Classification Models

Weak supervision is a machine learning (ML) approach that mitigates the challenges of creating large, annotated datasets for natural language processing (NLP). It involves using lower-accuracy or less detailed programmatically generated labels and limited labeled data in semisupervised learning [13-15]. This approach reduces the need for extensive manual labeling, speeding up the ML process and cutting costs. This method offers several advantages, including reduced annotation costs, improved scalability, and enhanced domain adaptability [13-15]. Weak supervision allows for the incorporation of domain expertise, supports iterative refinement, and enables model development in low-resource scenarios [13-15]. By minimizing manual labeling efforts, it facilitates the efficient creation of large-scale, domain-specific datasets, making it particularly valuable for developing robust NLP models across diverse fields [16-18].

Several recent studies have demonstrated the effectiveness of weak supervision in medical NLP tasks. Wang et al [19] combined this approach with pretrained word embeddings to create a rule-based NLP algorithm for the automatic generation of training labels. They evaluated the effectiveness of these auto-generated labels across 4 supervised learning architectures: support vector machine, random forest (RF), fully connected networks, and convolutional neural networks. Their comparative analysis demonstrated the versatility and efficacy of this auto-labeling technique as the generated labels performed successfully across diverse model frameworks [19]. Similarly, Cusick et al [18] applied weak supervision to address the challenge of exhaustive manual labeling in clinical contexts. Building on a previous study that used a rule-based NLP system to automatically label the clinical notes of 600 patients with potential suicidal ideation [18], they efficiently generated a sizable training dataset. This dataset was then used to train various statistical ML models and a convolutional neural network. Their research highlighted the potential of combining weak supervision and deep learning to enhance real-time clinical systems and facilitate research on suicidal ideation progression through automated analysis of clinical text [18]. In another study in this field, Fries et al [16] introduced Trove. This framework for weakly supervised medical entity classification leverages existing medical ontologies such as the Unified Medical Language System (UMLS) as a source of reusable, automated labeling heuristics. Trove’s key innovation is its use of a label model to learn the accuracies of individual ontologies and correct for label noise when combining multiple sources. The authors demonstrated a weakly supervised performance on named entity recognition tasks for chemicals, diseases, and drugs that achieved results within 1.3 to 4.9 *F*_1_-score points of fully supervised models.

These studies underscore the growing importance of weak supervision in overcoming the limitations of traditional manual annotation methods in clinical NLP tasks. By enabling the efficient creation of large-scale, domain-specific datasets, weak supervision is proving to be a valuable approach for developing robust NLP models across diverse clinical applications. This method offers a simplified way to auto-generate training labels, significantly reducing the need for manual annotation [16-19].

Our proposed method uniquely integrates high-accuracy expert labels with weak supervision, offering a more efficient and accurate clinical document classification solution that overcomes traditional techniques’ limitations. In the following section, we will describe the rationale, significance, and unique contributions of our proposed approach, demonstrating how it advances clinical NLP methods and addresses critical needs in the field.

### Rationale, Importance, and Contributions

NLP models designed for categorizing clinical documents can support various downstream AI or ML tasks in health care, such as risk prediction, clinical summarization, and probabilistic phenotyping [20-22]. The recent introduction of LLMs has created tremendous potential for health care to leverage these powerful tools. However, the bottleneck lies in the vast number of documents that these computationally intensive models need to process, which can reduce their efficiency and effectiveness. Lightweight NLP models solve this challenge by efficiently filtering documents and providing appropriate data inputs for more sophisticated models such as LLMs [10,23-25]. These lightweight models could potentially identify and select relevant documents, which may enhance the accuracy and efficiency of the overall AI or ML pipeline. However, the effectiveness of these models depends on input data quality. *International Classification of Diseases* (*ICD*) codes, traditionally used to identify relevant documents, have proven ineffective [26,27]. This observation underscores the limitations of relying exclusively on *ICD* codes for document selection in this context. The findings suggest the necessity for more refined and thorough identification methods to ensure the accurate capture of relevant COPD-related documents.

The potential effectiveness of these lightweight NLP models could stem from their ability to accurately identify and filter out irrelevant documents while potentially maintaining efficiency in computing power, memory use, and processing time. This efficiency is especially advantageous for resource-constrained health care systems, where simpler ML approaches that require significantly less computational power are often preferred over advanced NLP techniques [28] such as LLMs.

By leveraging heuristic training data and a small expert-annotated dataset, these models can be optimized with minimal labeled data, significantly reducing the manual annotation burden. This approach addresses the persistent challenge of limited annotated data in clinical settings, potentially accelerating the development and deployment of AI-driven health care solutions [19,29].

Our study makes 3 primary contributions to the field of clinical document classification. First, we introduce a novel framework that uniquely integrates high-accuracy expert labels with weak supervision for hyperparameter tuning, thus designing lightweight ML classifiers. This approach offers a more efficient and accurate solution for categorizing COPD-related documents as either “relevant” or “nonrelevant,” overcoming the limitations of traditional techniques. Second, we leveraged heuristic training data formulated by domain-expert clinicians to construct a training dataset without the need for extensive manual annotation. By using these expert hypotheses to generate “silver-standard” labels [30,31], we substantially mitigated the challenges commonly associated with manual annotation. Third, we conducted extensive experiments with diverse text representation techniques, rigorously evaluating the relative performance of different pipelines for identifying relevant documents while minimizing the inclusion of irrelevant ones.

Although our study focused on patients with COPD, the techniques we developed are designed to be adaptable across various clinical domains. Our approach of combining a small number of high-accuracy expert labels for hyperparameter tuning with a larger set of lower-accuracy labels for model training allows for potential improvements in model performance that may not be achievable using only lower-accuracy labels.

## Methods

### Overview

We constructed a retrospective observational cohort of patients with COPD attending Vanderbilt University Medical Center (VUMC) between January 1, 2012, and December 31, 2020. A series of NLP pipelines of all clinical narrative notes from this cohort were used to perform data evaluation. The objective of this study was to create an AI or ML pipeline using weak supervision with a small set of annotated data that could effectively determine the relevance of the notes for future AI or ML tasks for a clinical condition of interest (COPD). Our cohort inclusion criteria were patients with a diagnosis of COPD at any point during the study period and who were aged 40 years or older at the time of their diagnosis. COPD was defined based on administrative codes available from the electronic health record (EHR) for the *ICD, Ninth Revision, Clinical Modification* (491, 491.1, 491.2, 491.21, 491.22, 491.8, 491.9, 492, 492.8, 493.92, and 496) or *ICD, Tenth Revision, Clinical Modification* (J41.0, J41.1, J41.8, J42, J43.1, J43.2, J43.8, J43.9, J44.0, J44.1, and J44.9).

**Figure 1.**
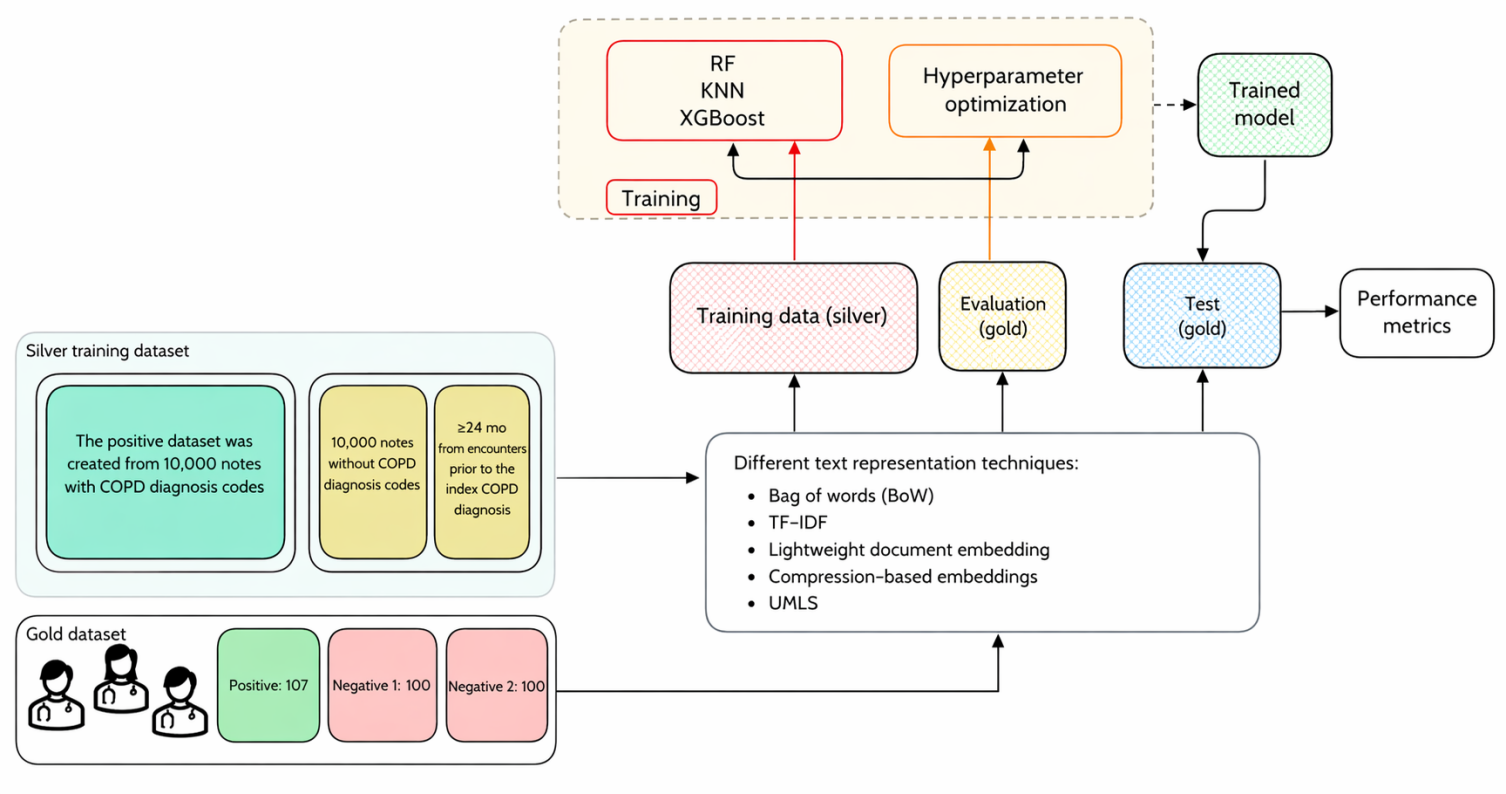
Framework for developing an effective lightweight classifier for clinical documents. BoW: bag of words; COPD: chronic obstructive pulmonary disease; KNN: k-nearest neighbor; RF: random forest; TF-IDF: term frequency–inverse document frequency; UMLS: Unified Medical Language System; XGBoost: extreme gradient boosting.

Figure 1 illustrates our NLP framework for classifying clinical notes related to COPD, including heuristic data creation, text representation, model training, optimization, and evaluation. Our heuristic training data, or “silver standard,” consisted of 20,000 clinical notes. We created this dataset using expert-defined rules: notes from encounters with COPD diagnosis codes were labeled as informative (positive), whereas those from encounters without COPD diagnosis codes or from 24 months or more prior to initial COPD diagnosis were labeled as noninformative (negative). This heuristic approach enabled the creation of a large training dataset without manual annotation, leveraging domain expertise to generate plausible labels. We used various text representation techniques to transform the free text into numerical features, including bag of words (BoW), term frequency–inverse document frequency (TF-IDF), lightweight document embedding, compression-based embeddings, and UMLS concept extraction. These representations fed into 3 ML classifiers: RF [32], k-nearest neighbor (KNN) [33], and extreme gradient boosting (XGBoost) [34]. For model optimization and evaluation, we used a separate gold-standard dataset manually annotated by clinical experts. We used a subset of these data for hyperparameter optimization, allowing us to fine-tune our models based on high-quality, expert-labeled data, and a held-out test set for final model assessment. By leveraging a large heuristically labeled dataset for training and then optimizing and testing with a carefully curated gold standard, this approach integrates heuristic-based training data creation, diverse text representation techniques, and a 2-stage evaluation process using expert-annotated data. The following subsections will explain each block in more detail.

### Ethical Considerations

This study was approved by the VUMC Institutional Review Board (IRB #210139) and received a waiver of informed consent as retrospective secondary use of routinely collected data. Data were stored and analyzed on secure, encrypted on-premises servers with restricted access. All study team members with access to data were trained in human participants research ethics. No participant compensation was provided. The manuscript and supplementary materials present summary information only and contain no identifiable patient information.

### Data Collection and Annotation

#### Overview

This subsection details our data collection and labeling methods for identifying COPD-relevant clinical notes. We used a 2-tiered strategy to build our training and test datasets: heuristic-based labeling for large-scale training (silver standard) and manual expert annotation for precise model optimization and testing (gold standard). This method effectively addresses the challenge in medical NLP of creating large amounts of reliable training data while ensuring thorough model evaluation.

We compiled a corpus of 10 million clinical notes extracted from 32 million raw notes collected over a decade (study period). From this collection, we developed our training and test datasets.

#### Heuristic Training Dataset (Silver Standard)

The training dataset consisted of positive and negative examples, identified using *ICD* code heuristics as follows:

1. The COPD encounter corpus (positive labels) consisted of 10,000 notes from encounters *ICD*-coded as COPD, likely containing COPD-relevant information.
2. The non-COPD encounter corpus (negative labels) consisted of 10,000 notes from patient encounters without any COPD diagnosis code, indicating content likely irrelevant to COPD.
3. The pre-COPD encounter corpus (negative labels) consisted of 10,000 notes from patient encounters occurring 24 months or more prior to the initial COPD diagnosis; this approach aimed to avoid including notes with early signs of COPD.

We constructed 3 training subsets to evaluate different heuristic combinations and their impact on classifier accuracy. Each subset contained 5000 positive examples from (1) and 5000 negative examples. The negative examples varied across subsets: the first used a mix of 2500 each from (2) and (3), the second used 5000 from (2) only, and the third used 5000 from (3) only.

This design allowed us to assess which combination produced the most accurate classifier. We ensured diversity in patient demographics, timing, and note types across all samples.

#### Test Data (Gold Standard)

Clinical subject matter experts developed an annotation guideline to determine the relevance of documents for COPD diagnosis and exacerbation prediction. The guideline was iteratively refined through consultations with clinical experts to ensure consistent labeling criteria. Using this guideline, we created a reference standard for evaluation by randomly selecting 307 documents from the positive and negative training datasets, including 107 COPD-coded notes, 100 non-COPD encounter notes, and 100 pre-COPD encounter notes. Three clinicians independently reviewed and annotated these documents, labeling them as either relevant or irrelevant for COPD exacerbation prediction. Disagreements were resolved through adjudication among the reviewers to produce the final consensus labels. The annotated documents were then divided into a validation set used for model optimization during development and a held-out test set used for final evaluation. Specifically, 84 documents were used for validation, whereas 223 documents were reserved as the independent test set. The final test set size reflected the adjudicated label set after reconciliation of annotations among the 3 reviewing clinicians.

### NLP Model Development

#### Overview

The development of the NLP pipeline for classifying clinical documents related to COPD diagnosis involved several key stages. Initially, various NLP techniques were applied to transform clinical text into informative numerical representations. These numerical representations served as input for ML models, which were subsequently optimized to enhance performance. Finally, the fully optimized models were assessed against a held-out test set to measure their effectiveness. The following subsections will explain in detail the different text representation methods and ML approaches and the processes of training, optimization, and testing the models.

#### Text Representation

This step involved exploring various text representation methods, including BoW, TF-IDF, lightweight document embedding, and lossless compression representation.

BoW is a text representation technique that treats each document as a collection of words, disregarding their order. It creates a vector where each element corresponds to the frequency of a word in the document, ignoring context [35].

TF-IDF is a text analysis method that evaluates the importance of words in a document within a larger corpus. It calculates a score for each word based on its frequency in the document and its rarity across all documents, helping identify key terms [36].

Lightweight document embedding [37,38] is a modern text representation approach that uses pretrained language models such as Word2Vec or FastText to convert words or phrases into dense, continuous-valued vectors. These embeddings capture semantic relationships between words and are useful for various NLP tasks. Using distributed memory and distributed BoW approaches for enhancing paragraph and document embeddings [38], we incorporated the hierarchical softmax algorithm [39].

Compression representation uses lossless compression techniques. The key idea is to leverage compressors to efficiently represent text information as they excel at capturing regularity. Normalized compression distance [40,41] quantifies shared information between text objects by comparing their compressed lengths, making it suitable for classification. Using gzip as the compressor, this lightweight and universal method calculates normalized compression distances between testing and training data for KNN classification, offering a simple and resource-efficient alternative to deep neural networks for text classification tasks. This lightweight methodology is readily applicable to extensive text and has demonstrated better performance compared to the bidirectional encoder representations from transformers model [41]. While this approach has been tested on general texts such as news articles and Yahoo Answers, we extended its use by applying it to clinical documents. However, ML classifiers such as RF and XGBoost do not natively use compression distance metrics as features. Thus, we transformed raw text via compression into the gzip format. The length of the compressed data then served as the key predictive feature for RF and XGBoost models.

UMLS text representation is a comprehensive resource developed by the National Library of Medicine to facilitate the integration of biomedical information systems [42,43]. One of its key components is the representation of biomedical concepts and their relationships through various text formats. In UMLS, each concept is assigned a unique identifier called a Concept Unique Identifier (CUI). CUIs are alphanumeric codes that remain stable across different versions of the UMLS, serving as a means to identify and link concepts across multiple vocabularies and ontologies. For example, the CUI C0018787 represents the concept of “heart” in the UMLS. In this representation, text documents were encoded as vectors where each element corresponded to a unique CUI. The presence of a CUI was indicated by a nonzero value, which also encoded contextual information such as assertion status (eg, positive or negative). This approach combines elements of one-hot encoding with additional semantic information, allowing for a more nuanced representation of the document’s content.

#### ML Training, Optimization, and Evaluation Metrics

This study used a rigorous methodology to train, optimize, and compare 3 ML algorithms: RF, XGBoost, and KNN. We used a weakly supervised learning approach, training models on silver-labeled documents to learn discriminative features for identifying clinically relevant notes without extensive manual annotation. While not perfect, this silver-labeled dataset provided a valuable starting point for model training.

The optimization process started with hyperparameter tuning through grid search, systematically examining key parameters for each model. For RF, we considered the number of trees, maximum depth, and minimum sample split. XGBoost optimization focused on learning rate, maximum depth, subsample, and number of boosting rounds. For KNN, we tuned the number of neighbors and distance metric. Five-fold cross-validation assessed each hyperparameter combination, with the *F*_1_-score serving as the primary metric due to its capacity to balance precision and recall.

To refine our approach, we leveraged a small set of clinician-annotated evaluation data. This allowed for better fine-tuning of the models to distinguish document relevance, balancing the primary aim of maximizing recall (to ensure identification of as many truly relevant documents as possible) with maintaining precision (to avoid excessive misclassification of irrelevant documents). We proceeded to fine-tune the models by adjusting classification thresholds. Probability predictions were generated on a validation dataset, with thresholds ranging from 0.0 to 1.0 in 0.1 increments. Precision, recall, and *F*_1_-score were calculated at each threshold, facilitating the identification of an optimal threshold that maximized the *F*_1_-score while maintaining an appropriate balance between precision and recall. The final evaluation was conducted on a held-out test set, focusing primarily on key metrics: recall (sensitivity), precision (positive predictive value), specificity, and *F*_1_-score for the relevant class. Recall measures the fraction of total relevant documents correctly classified, whereas precision assesses the accuracy in identifying only relevant documents, minimizing false positives [44]. Specificity evaluates the system’s ability to correctly identify nonrelevant documents, providing a comprehensive picture of classification performance. The *F*_1_-score, as the harmonic mean of precision and recall, offers a combined measure of classification accuracy [44]. Detailed performance metrics at various classification thresholds for the best-performing model are provided in Multimedia Appendix 1, offering deeper insights into model behavior across diverse scenarios. The 95% CIs for *F*_1_-scores were computed using bootstrap resampling (1000 iterations) on the held-out test set, providing robust uncertainty estimates for all reported performance metrics.

### Experiment Design

#### Baseline

The baseline classification for COPD-related documents used expert-defined heuristics based on *ICD* codes and the temporal relevance of notes. These heuristics were evaluated against a manually annotated gold-standard test dataset, which included examples from each category (COPD coded, non-COPD coded, and pre-COPD temporal). This evaluation served 2 purposes: first, to assess the effectiveness of these simple rules in identifying COPD-relevant notes compared to clinician annotations and, second, to establish a baseline for comparison with ML approaches. The subsequent ML models were trained on the heuristically labeled data, optimized using a small annotated dataset, and then tested on the same gold-standard test data. This process determined whether ML could optimize the classification process while requiring only a small annotated dataset for fine-tuning.

#### Experiment Setup

We experimented with 3 classifiers—RF, XGBoost, and KNN—using BoW representations as these models are known to effectively handle various data types [45,46]. Following the same standardized procedure, we also experimented with the ML models using other text representations: TF-IDF, lightweight document embeddings, and compression-based features.

#### Hyperparameter Tuning

Hyperparameter tuning was conducted for the RF, XGBoost, and KNN classifiers to optimize their performance on the text classification task. For RF, we explored the number of trees (100-500), maximum depth (10-30 and none), minimum samples for split and leaf nodes (2-10 and 1-4, respectively), feature selection strategies for node splitting, and class weight options. XGBoost tuning focused on learning rate (0.01-0.3), number of estimators (100-1000), maximum depth (3-10), minimum child weight (1-6), subsampling ratio (0.5-1.0), and column sampling by tree (0.5-1.0). For KNN, we varied the number of neighbors (1-20), weighting function (uniform and distance), distance metrics (Euclidean, Manhattan, and Minkowski), and leaf size (10-50). Grid search with 5-fold cross-validation was used for all models using multiple scoring metrics (*F*_1_-score, precision, recall, and specificity) to evaluate performance. This approach allowed for the selection of optimal hyperparameter combinations that balanced model complexity, computational efficiency, and generalization performance tailored to the specific challenges of text classification.

#### Threshold Analysis

In our case, optimizing recall was crucial, particularly because the cost of false negatives was high. While classification models often default to a 0.5 threshold for converting probability predictions into class labels, it was possible that this threshold would not be ideal for our needs. Our threshold analysis evaluated model performance across a range of thresholds, from 0.0 to 1.0 in increments of 0.1. At each threshold, we calculated precision, recall, specificity, and the *F*_1_-score. This analysis allowed us to fine-tune the model’s decision boundary to maximize recall while maintaining excellent precision, ensuring that we identified as many relevant cases as possible without significantly increasing the number of false positives.

For all experiments, models were optimized on a separate validation set to find the optimal hyperparameters that maximized recall as accurately identifying all relevant documents was crucial. Instead of relying solely on the default threshold, we iteratively adjusted the recall threshold to find the optimal cutoff for each model. The optimized parameters based on this evaluation were then used in the final models and applied to the held-out test set. By adhering to this consistent methodology, we were able to fairly assess the performance of different classifiers and text representations.

## Results

### Baseline Performance

**Figure 2.**
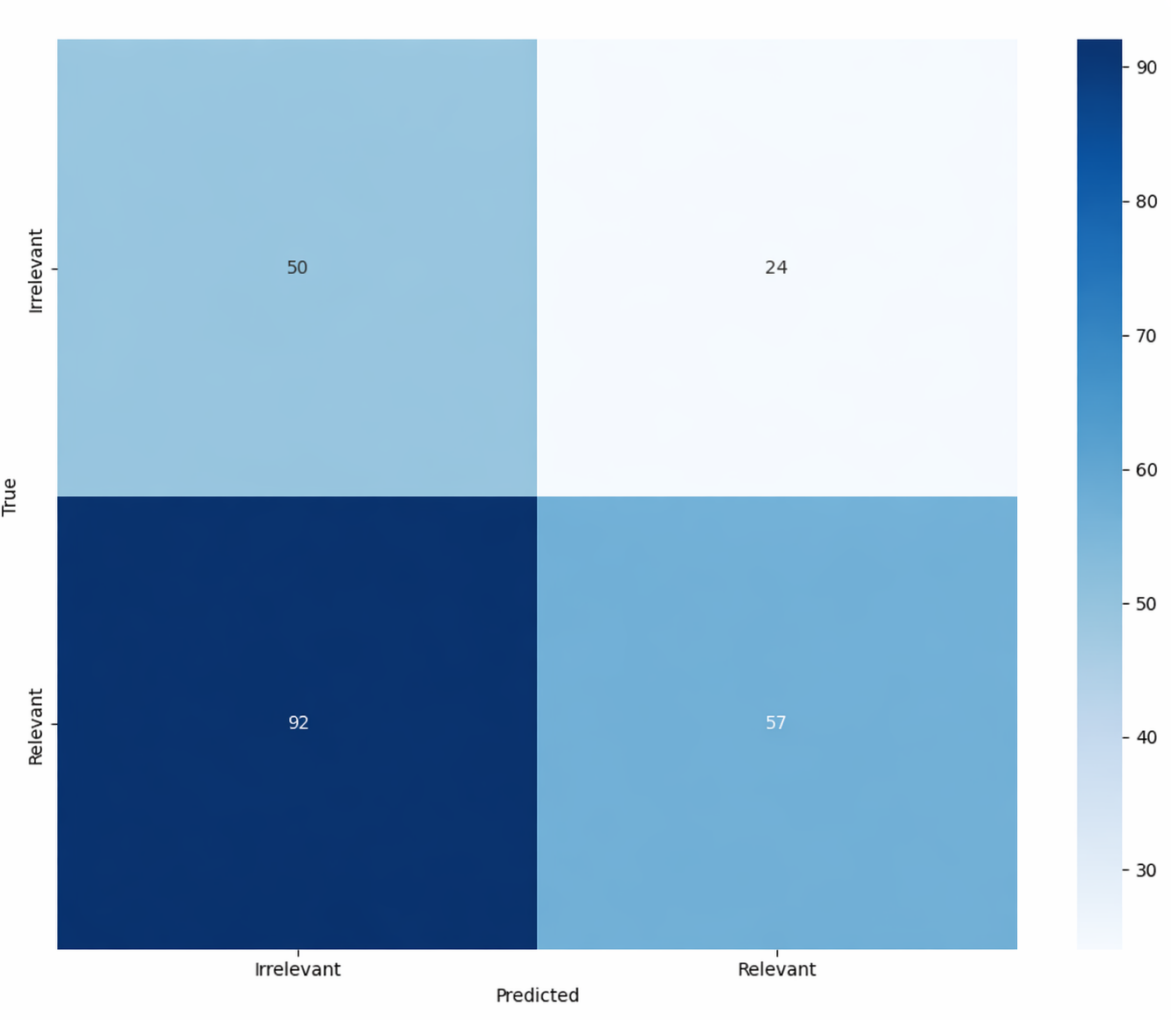
Baseline confusion matrix on the test data.

The defined *ICD*-based heuristics were evaluated against manually annotated gold-standard test data. Table 1 shows the baseline model’s performance in classifying COPD-related documents. Figure 2 shows the baseline confusion matrix on the test data. The heuristics correctly identified 50 irrelevant and 57 relevant documents while misclassifying 24 irrelevant and 92 relevant documents.

**Table 1.** The baseline model’s performance in classifying chronic obstructive pulmonary disease–related documents.

	Precision (PPV^a^)	Recall (sensitivity)	Specificity	*F*_1_-score (95% CI)
Baseline model	0.70	0.38	0.68	0.50 (0.43-0.56)

a PPV: positive predictive value.

### ML Framework Performance

The results validate the effectiveness of our framework, which used various text representation techniques and ML models to eliminate irrelevant documents and prioritize information in relevant ones. We found that combining both negative cases (non-COPD coded and pre-COPD temporal) consistently produced the best model across all representation techniques. As shown in Tables S1 to S5 in Multimedia Appendix 1, the mixed-negative configuration yielded the highest *F*_1_-score across most text representations, supporting this choice as the primary training strategy. Tables S1 to S5 in Multimedia Appendix 1 provide comprehensive results for all combinations, but here, we focus on the results obtained by training the model on the combined dataset using both negative heuristics.

Table 2 presents a comparative analysis of various ML models (RF, XGBoost, and KNN) across different document representations. The RF model consistently demonstrated superior performance with balanced precision, recall, and *F*_1_-score for the relevant class when using BoW representation. In the TF-IDF representation, KNN showed high precision but lower recall, whereas RF provided a more balanced performance. With lightweight document embeddings, RF achieved high recall but lower precision for the irrelevant class, indicating a trade-off. For compression-based representations, RF and XGBoost performed comparably, with KNN achieving a higher *F*_1_-score. Finally, in the UMLS representation, XGBoost marginally outperformed RF in precision and *F*_1_-score, whereas KNN showed high precision but lower recall.

**Table 2.** Performance comparison of classification models on the chronic obstructive pulmonary disease dataset using various representations.

Representation and model	Precision (PPV^a^)	Recall (sensitivity)	Specificity	*F*_1_-score (95% CI)
BoW^b^				
RF^c^	0.77	0.71	0.57	0.74 (0.7-0.78)
XGBoost^d^	0.75	0.67	0.54	0.71 (0.65-0.76)
KNN^e^	0.71	0.47	0.62	0.57 (0.53-0.62)
TF-IDF^f^				
RF	0.76	0.69	0.57	0.73 (0.68-0.78)
XGBoost	0.71	0.64	0.46	0.67 (0.61-0.71)
KNN	0.80	0.37	0.81	0.51 (0.46-0.55)
Lightweight document embedding				
RF	0.73	0.86	0.36	0.80 (0.76-0.87)
XGBoost	0.73	0.74	0.45	0.74 (0.71-0.77)
KNN	0.89	0.32	0.92	0.47 (0.43-0.52)
Compression based				
RF	0.78	0.45	0.74	0.57 (0.52-0.6)
XGBoost	0.78	0.45	0.74	0.57 (0.54-0.62)
KNN	0.75	0.64	0.57	0.69 (0.63-0.74)
UMLS^g^				
RF	0.74	0.55	0.64	0.63 (0.58-0.69)
XGBoost	0.76	0.56	0.46	0.65 (0.6-0.68)
KNN	0.86	0.30	0.86	0.44 (0.38-0.51)

a PPV: positive predictive value.

b BoW: bag of words.

c RF: random forest.

d XGBoost: extreme gradient boosting.

e KNN: k-nearest neighbor.

f TF-IDF: term frequency–inverse document frequency.

g UMLS: Unified Medical Language System.

Lightweight document embedding combined with the RF model was the most effective method for extracting COPD-relevant documents. This approach achieved an *F*_1_-score of 0.80 (95% CI 0.76-0.87) for the relevant class, outperforming other representation techniques examined in this study.

### Best-Performing Model Comparison and Threshold Analysis

**Figure 3.**
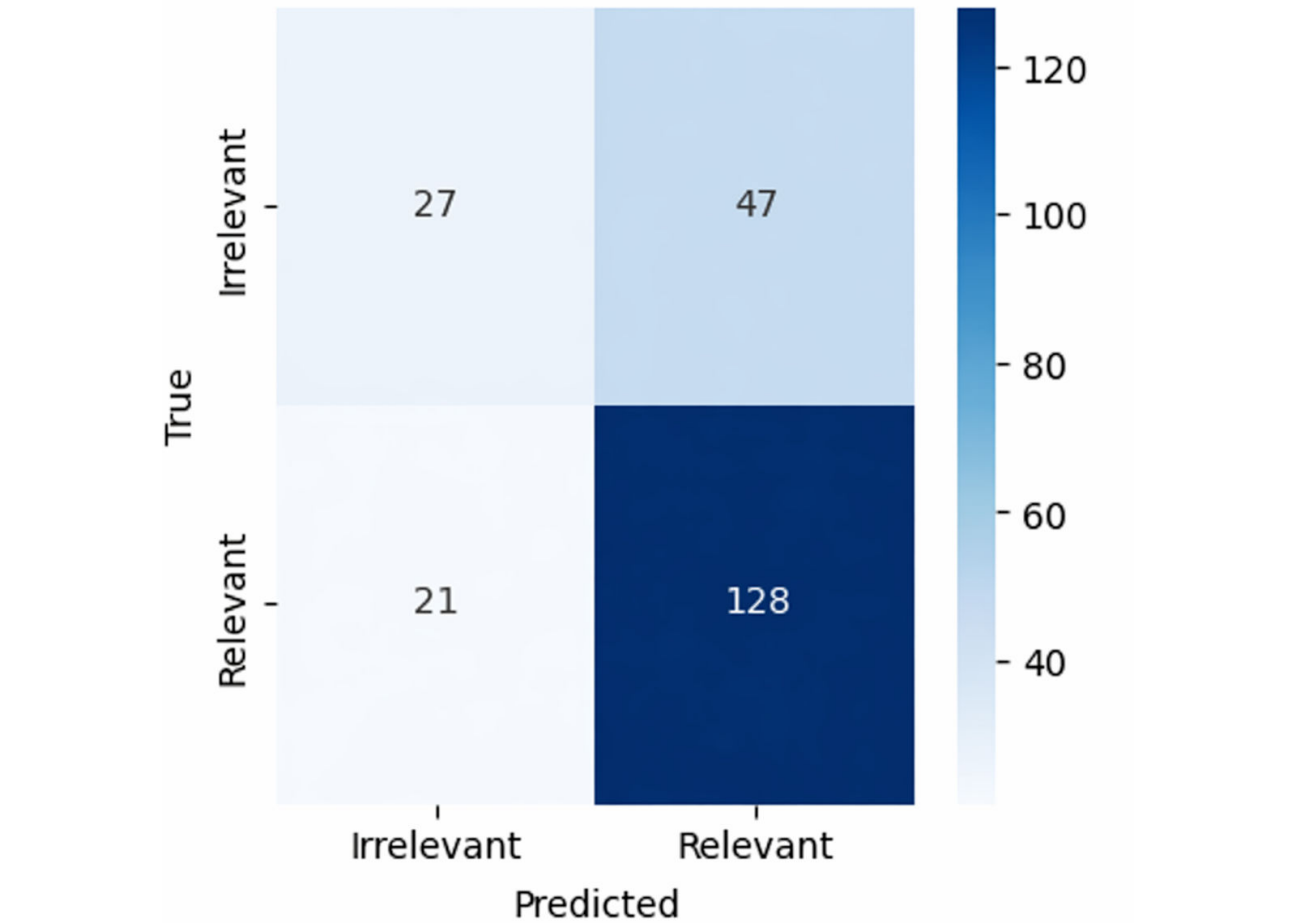
Best-performing natural language processing pipeline confusion matrix.

The baseline model confusion matrix (Figure 2) correctly identified 50 irrelevant and 57 relevant documents, with 24 irrelevant and 92 relevant documents misclassified. The best-performing NLP pipeline confusion matrix (Figure 3) correctly identified 27 irrelevant and 128 relevant documents, with 47 irrelevant and 21 relevant documents misclassified. The best-performing model significantly improved relevant document identification (128 vs 57) and reduced relevant document misclassification (21 vs 92).

Although the best-performing NLP pipeline had a higher irrelevant document misclassification rate (as highlighted by low specificity), this trade-off was acceptable given the optimization for sensitivity, which was the primary goal. The detailed results for the best-performing classifier at various cutoff points are shown in Figure S1 in Multimedia Appendix 1. The figure illustrates the relationship among precision, sensitivity, and specificity across different thresholds for the classification model.

## Discussion

### Principal Findings

Our study demonstrated the effectiveness of lightweight ML models, particularly an RF classifier with lightweight document embeddings, in extracting COPD-relevant documents from EHRs. This approach significantly outperformed baseline heuristic methods, achieving high recall (0.86) and *F*_1_-score (0.80, 95% CI 0.76‐0.87) for relevant documents. These findings represent a significant advancement in efficient EHR processing, offering a scalable solution for filtering large volumes of clinical documents in resource-constrained environments. By improving the identification of relevant information, this approach has the potential to enhance the performance of clinical decision support systems and optimize resource use in health care applications.

### ML Optimization Over Heuristic Approaches

Traditional heuristic approaches, such as relying on *ICD* codes for document filtering, have proven inadequate in accurately capturing relevant clinical information. Our findings confirm that a substantial proportion of documents with COPD *ICD* codes lack significant relevant information (as illustrated in Figure 2), highlighting the limitations of such methods. In contrast, our ML framework effectively distinguished relevant from irrelevant documents (as illustrated in Figure 3), thereby improving the sensitivity and identification of relevant documents. ML models outperformed baseline approaches by optimizing small annotations, learning deeper patterns, adapting flexibly, generalizing better from imperfect examples, handling noisy data robustly, and using advanced feature extraction techniques [16,47].

### Improvement and Effectiveness of the Model

A primary contribution of our work is demonstrating how lightweight models such as an RF classifier paired with lightweight document embeddings can achieve high performance with minimal annotation data. By using weak supervision, we generated “silver-standard” labels from domain-expert heuristics, significantly reducing the need for extensive manual annotation. This method not only streamlined the training process but also enhanced the model’s scalability across different clinical domains.

Our methodology uniquely integrated a large corpus of weakly supervised silver-standard labels for initial training with a small set of high-fidelity gold-standard labels for hyperparameter optimization. This approach not only streamlined the training process but also enhanced model performance and generalizability across diverse clinical domains.

As shown in Table 2, among the evaluated text representation techniques, lightweight document embedding coupled with the RF classifier exhibited superior performance in classifying relevant COPD documents, achieving a recall of 0.86 and an *F*_1_-score of 0.80 (95% CI 0.76-0.87) for the relevant class. This performance significantly surpassed the baseline model’s *F*_1_-score of 0.50 (95% CI 0.43-0.56), underscoring the efficacy of our approach compared to conventional heuristic methods.

The incorporation of a limited set of high-fidelity labels for hyperparameter tuning resulted in a substantial improvement in model performance. The model-agnostic nature of this approach rendered it applicable to any classifier requiring hyperparameter optimization.

Additionally, the results underscore the critical role of text representation in model performance. While both RF and XGBoost proved to be robust classifiers, their effectiveness was notably influenced by the choice of text representation technique. For instance, RF’s *F*_1_-score for the relevant class ranged from 0.57 (95% CI 0.52-0.60) with compression-based representation to 0.80 (95% CI 0.76-0.87) with lightweight document embedding.

### Limitations and Future Directions

While our study demonstrated the effectiveness of lightweight document embedding and ML models in extracting relevant COPD documents, several limitations should be considered. First, the dataset used in this study was specific to COPD, and the generalizability of our findings to other clinical conditions remains to be evaluated. Future research should examine the applicability of this framework across a broader range of diseases and clinical document types. Second, the data were derived from a single institution (VUMC), which may limit external validity due to variations in clinical documentation practices across health care systems. Multi-institutional validation using datasets from different health systems would help assess the robustness and portability of the proposed framework. Finally, while this study focused on binary classification of documents as relevant or irrelevant, future work could explore more granular categorization of clinical documents through multi-class classification (eg, exacerbation-related notes, diagnostic documentation, and treatment updates). Such extensions could further enhance the utility of this framework for downstream clinical decision support and LLM-based information extraction pipelines.

### Conclusions

This study presents a novel framework for extracting COPD-relevant clinical documents using lightweight document embedding and ML models. Our approach effectively identifies relevant documents while minimizing the identification of irrelevant ones, enhancing the quality of information for clinical decision support systems and improving patient outcomes. Future research should explore the generalizability of our findings to other chronic conditions and integrate this model into predictive analytics for greater efficiency and effectiveness. This framework can be adopted across diverse health care domains, contributing to improved patient care and clinical outcomes.

## Supplementary material

10.2196/84326 Multimedia Appendix 1 Supplementary Tables 1 through 5 present performance comparisons for random forest, extreme gradient boosting, and k-nearest neighbors models on a chronic obstructive pulmonary disease dataset. These evaluations incorporate bag of words, term frequency-inverse document frequency, lightweight document embeddings, compression-based, and Unified Medical Language System representations. Supplementary Figure 1 illustrates variations in precision, sensitivity, and specificity across different thresholds.

## Abbreviations

AI: artificial intelligence
BoW: bag of words
COPD: chronic obstructive pulmonary disease
CUI: Concept Unique Identifier
EHR: electronic health record
ICD: International Classification of Diseases
KNN: k-nearest neighbor
LLM: large language model
ML: machine learning
NLP: natural language processing
RF: random forest
TF-IDF: term frequency–inverse document frequency
UMLS: Unified Medical Language System
VUMC: Vanderbilt University Medical Center
XGBoost: extreme gradient boosting
