# Supplementary material for: Scalable Identification of Clinically Relevant Chronic Obstructive Pulmonary Disease Documents in Large-Scale Electronic Health Record Datasets With a Lightweight Natural Language Processing Model: Retrospective Cohort Study
Source: JMIR Med Inform. 2026 May 12;14:e84326. doi: 10.2196/84326 (PMC13167063; doi:10.2196/84326)
Supplement: Multimedia Appendix 1 [file medinform-v14-e84326-s001.docx]

# Supplementary Materials

Table S1. Performance Comparison of Classification Models on COPD Dataset using BoW Representation

| 5,000 positives + 5000 of an equal combination of both negative sets (B and C) | Models | Precision | Recall | Specificity | F1-score |
| --- | --- | --- | --- | --- | --- |
|  | RF | 0.77 | 0.71 | 0.57 | 0.74 |
|  | XGBOOST | 0.75 | 0.67 | 0.54 | 0.71 |
|  | KNN | 0.71 | 0.47 | 0.62 | 0.57 |
|  | **Models** | **Precision** | **Recall** | **Specificity** | **F1-score** |
| 5,000 positive + 5,000 negatives (temporal) | RF | 0.68 | 0.70 | 0.42 | 0.69 |
|  | XGBOOST | 0.70 | 0.69 | 0.45 | 0.70 |
|  | KNN | 0.68 | 0.42 | 0.55 | 0.52 |
| 5,000 positives + 5,000 negative B set | **Models** | **Precision** | **Recall** | **Specificity** | **F1-score** |
|  | RF | 0.72 | 0.69 | 0.50 | 0.70 |
|  | XGBOOST | 0.72 | 0.65 | 0.49 | 0.68 |
|  | KNN | 0.69 | 0.40 | 0.58 | 0.51 |

Table S2. Performance Comparison of Classification Models on COPD Dataset using TF-IDF Representations

| 5,000 positives + 5000 of an equal combination of both negative sets (B and C) | Models | Precision | Recall | Specificity | F1-score |
| --- | --- | --- | --- | --- | --- |
|  | RF | 0.76 | 0.69 | 0.57 | 0.73 |
|  | XGBOOST | 0.71 | 0.64 | 0.46 | 0.67 |
|  | KNN | 0.80 | 0.37 | 0.81 | 0.51 |
|  | **Models** | **Precision** | **Recall** | **Specificity** | **F1-score** |
| 5,000 positives + 5,000 negative B set | RF | 0.71 | 0.60 | 0.45 | 0.60 |
|  | XGBOOST | 0.69 | 0.62 | 0.42 | 0.65 |
|  | KNN | 0.76 | 0.36 | 0.63 | 0.49 |
| 5,000 positives + 5,000 negative C set | **Models** | **Precision** | **Recall** | **Specificity** | **F1-score** |
|  | RF | 0.76 | 0.69 | 0.57 | 0.72 |
|  | XGBOOST | 0.71 | 0.63 | 0.42 | 0.66 |
|  | KNN | 0.78 | 0.33 | 0.68 | 0.47 |

Table S3 Performance Comparison of Classification Models on COPD using Lightweight Document Embedding Representation

| 5,000 positives + 5000 of an equal combination of both negative sets (B and C) | Model | Precision | Recall | Specificity | F1-score |
| --- | --- | --- | --- | --- | --- |
|  | RF | 0.73 | 0.86 | 0.36 | 0.80 |
|  | XGBoost | 0.73 | 0.74 | 0.45 | 0.74 |
|  | KNN | 0.89 | 0.32 | 0.92 | 0.47 |
|  | **Models** | **Precision** | **Recall** | **Specificity** | **F1-score** |
| 5,000 positives + 5,000 negative B set | RF | 0.70 | 0.77 | 0.41 | 0.73 |
|  | XGBoost | 0.69 | 0.72 | 0.43 | 0.70 |
|  | KNN | 0.84 | 0.30 | 0.88 | 0.44 |
| 5,000 positives + 5,000 negative C set | **Models** | **Precision** | **Recall** | **Specificity** | **F1-score** |
|  | RF | 0.71 | 0.81 | 0.40 | 0.76 |
|  | XGBoost | 0.70 | 0.74 | 0.44 | 0.72 |
|  | KNN | 0.86 | 0.31 | 0.90 | 0.46 |

Table S4 Performance Comparison of Classification Models on COPD using Compression-based Representation.

| 5,000 positives + 5000 of an equal combination of both negative sets (B and C) | Models | Precision | Recall | Specificity | F1-score |
| --- | --- | --- | --- | --- | --- |
|  | RF | 0.78 | 0.45 | 0.74 | 0.57 |
|  | XGBOOST | 0.78 | 0.45 | 0.74 | 0.57 |
|  | KNN | 0.75 | 0.64 | 0.57 | 0.69 |
|  | **Models** | **Precision** | **Recall** | **Specificity** | **F1-score** |
| 5,000 positives + 5,000 negative B set | RF | 0.62 | 0.41 | 0.63 | 0.49 |
|  | XGBOOST | 0.71 | 0.47 | 0.71 | 0.57 |
|  | KNN | 0.68 | 0.57 | 0.52 | 0.62 |
| 5,000 positives + 5,000 negative C set | **Models** | **Precision** | **Recall** | **Specificity** | **F1-score** |
|  | RF | 0.76 | 0.42 | 0.71 | 0.54 |
|  | XGBOOST | 0.73 | 0.28 | 0.72 | 0.41 |
|  | KNN | 0.69 | 0.60 | 0.5 | 0.64 |

Table S5 Performance Comparison of Classification Models on COPD using UMLS Representation.

| 5,000 positives + 5000 of an equal combination of both negative sets (B and C) | Models | Precision | Recall | Specificity | F1-score |
| --- | --- | --- | --- | --- | --- |
|  | **RF** | 0.74 | 0.55 | 0.64 | 0.63 |
|  | XGBOOST | 0.76 | 0.56 | 0.46 | 0.65 |
|  | KNN | 0.86 | 0.30 | 0.86 | 0.44 |
|  | **Models** | **Precision** | **Recall** | **Specificity** | **F1-score** |
| 5,000 positives + 5,000 negative B set | **RF** | 0.69 | 0.51 | 0.62 | 0.59 |
|  | XGBOOST | 0.70 | 0.51 | 0.42 | 0.59 |
|  | KNN | 0.80 | 0.25 | 0.76 | 0.38 |
| 5,000 positives + 5,000 negative C set | **Models** | **Precision** | **Recall** | **Specificity** | **F1-score** |
|  | **RF** | 0.64 | 0.45 | 0.54 | 0.53 |
|  | XGBOOST | 0.72 | 0.46 | 0.42 | 0.56 |
|  | KNN | 0.82 | 0.25 | 0.72 | 0.38 |


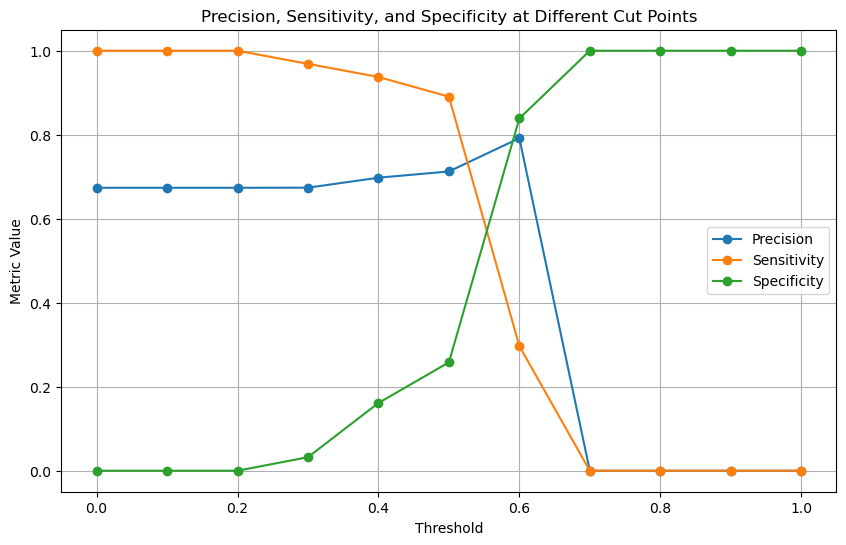


Figure S1: Variation of Precision, Sensitivity, and Specificity Across Different Thresholds
